# 2421 Development and validation of a translational rat model of neonatal abstinence syndrome

**DOI:** 10.1017/cts.2018.63

**Published:** 2018-11-21

**Authors:** Lisa Brents, Bryce A. Griffin, Caitlin Caperton, Lauren Russell, Christian Cabanlong, Catheryn Wilson, Kyle Urquhart, Brad Martins, Amy L. Patton, Alexander W. Alund, S. Michael Owens, William E. Fantegrossi, Jeffery H. Moran

**Affiliations:** 1 PinPoint Testing, LLC and University of Arkansas for Medical Sciences; 2 University of Arkansas Translational Research Institute

## Abstract

OBJECTIVES/SPECIFIC AIMS: Rodent models can be used to study neonatal abstinence syndrome (NAS), but the applicability of findings from the models to NAS in humans is not well understood. The objective of this study was to develop a rat model of norbuprenorphine-induced NAS and validate its translational value by comparing blood concentrations in the norbuprenorphine-treated pregnant rat to those previously reported in pregnant women undergoing buprenorphine treatment. METHODS/STUDY POPULATION: Pregnant Long-Evans rats were implanted with 14-day osmotic minipumps containing vehicle, morphine (positive control), or norbuprenorphine (0.3–3 mg/kg/d) on gestation day 9. Within 12 hours of delivery, pups were tested for spontaneous or precipitated opioid withdrawal by injecting them with saline (10 mL/kg, i.p.) or naltrexone (1 or 10 mg/kg, i.p), respectively, and observing them for well-validated neonatal withdrawal signs. Blood was sampled via indwelling jugular catheters from a subset of norbuprenorphine-treated dams on gestation day 8, 10, 13, 17, and 20. Norbuprenorphine concentrations in whole blood samples were quantified using LC/MS/MS. RESULTS/ANTICIPATED RESULTS: Blood concentrations of norbuprenorphine in rats exposed to 1–3 mg/kg/d of norbuprenorphine were similar to levels previously reported in pregnant women undergoing buprenorphine treatment. Pups born to dams treated with these doses exhibited robust withdrawal signs. Blood concentrations of norbuprenorphine decreased across gestation, which is similar to previous reports in humans. DISCUSSION/SIGNIFICANCE OF IMPACT: These results suggest that dosing dams with 1–3 mg/kg/day norbuprenorphine produces maternal blood concentrations and withdrawal severity similar to those previously reported in humans. This provides evidence that, at these doses, this model is useful for testing hypotheses about norbuprenorphine that are applicable to NAS in humans.

